# Holmium Laser Enucleation of Prostate: Is novel En Bloc Enucleation Technique Better Than the Traditional 2-Lobe Technique—A Prospective Randomized Study

**DOI:** 10.5152/tud.2024.23177

**Published:** 2024-01-01

**Authors:** Abhay Dinkar Mahajan, Arpit Rajendra Sharma, Martand G Patil

**Affiliations:** MGM Medical College Aurangabad, MGM Institute of Health Sciences, Navi Mumbai, India

**Keywords:** Enucleation, holmium, laser, prostate, stress urinary incontinence

## Abstract

**Objective:**

To compare the safety and efficacy of the en bloc technique with the standard 2-lobe technique for holmium laser enucleation of the prostate (HoLEP).

**Methods:**

This prospective study included patients with benign prostatic hyperplasia (BPH) who underwent HoLEP from September 2020 to March 2022, by en bloc or 2-lobe technique. Patient demographics, prostate volume, enucleation, morcellation and operative time, and incidence of postoperative incontinence were compared between the 2 groups.

**Results:**

We included 64 patients (30 en bloc and 34 2-lobe techniques) who underwent HoLEP in this study. The mean age, prostate volume, creatinine, and PSA of patients were comparable in both groups [(68.53 vs. 67.55 years; *P* = .62), (79.43 vs. 79.88 g, *P* = .92), (1.08 mg/dL vs. 1.20 mg/dL, *P* = .35), (3.78 vs. 4.63 ng/mL; *P* = .376), respectively]. The enucleation time was significantly shorter in the en bloc group than in the 2-lobe group (54.2 vs. 61.67; *P* = .03). Additionally, the mean operative time was also comparatively shorter in the en bloc group than the 2-lobe group (72.36 vs. 80.50; *P* = .057). The improvement in the quality-of-life (QoL) score was significantly better with en bloc than the 2-lobe group (3.80 vs. 2.11; *P* = .01). There was a significant difference in stress urinary incontinence on days 1, 7, and 30 (*P* < .001) with en bloc compared to the two-lobe technique.

**Conclusion:**

Although the outcomes of en bloc and 2-lobe endoscopic enucleation of prostate techniques were comparable, the en bloc technique seems to be a better option in most patients undergoing HoLEP due to less enucleation and operative time and lowered stress urinary incontinence incidence.

**Main points**

- Anatomical endoscopic enucleation of the prostate with holmium laser is a safe and effective procedure for medium- to large-sized prostates.
- En bloc enucleation has shorter enucleation and operative times as compared to the standard 2-lobe technique.
- The improvement in the QoL score is better with the en bloc technique than the two-lobe technique.
- The incidence of stress urinary incontinence is significantly lower in the en bloc technique as compared to the 2-lobe technique.

## Introduction

At present, the guidelines by the American Urological Association and the European Association of Urology suggest endoscopic enucleation of the prostate (EEP) as the treatment modality for patients with benign prostatic hyperplasia (BPH).^1,2^ To achieve results consistent with the electrosurgical techniques in BPH, multiple laser-based minimally invasive treatment alternatives like holmium laser, thulium laser, and potassium titanyl phosphate laser have been used.^3-5^ Following its first brief by Gilling in 1998, holmium laser enucleation of the prostate (HoLEP) became the most preferred treatment modality for the treatment of BPH,^6^ mainly due to its higher efficacy compared to the standard transurethral resection of the prostate.^7,8^ In addition, low morbidity, short hospital stay, and less catheter duration make HoLEP a safer and more effective treatment than open prostatectomy in large prostate.^9^ Holmium laser enucleation of the prostate was initially performed using a 2- or 3-lobe approach that involved an initial incision at 5 and 7 o’clock and the removal of the median lobe, if present.^10^ Various surgeons later made multiple alterations to this process. In 2015, a study by Sancha et al^11^ described the en bloc technique of EEP with a green light laser. When the urologists were well acquainted with the anatomy of the surgical planes (prostatic capsule and adenoma) and when multiple surgical alterations were published, the en bloc technique became prominent. In 2017, Saredi et al^4^ introduced the en bloc technique of EEP with thulium laser. A study by Ito et al compared the efficacy of the complete en bloc technique with the traditional 3-lobe technique.^14^ In 2019, Saitta et al^13^ observed better results in the form of enhanced visualization, rapid results, correct surgical planes, and intact sphincter function with the en bloc early apical release procedure of EEP.

In this study, EEP with HoLEP was initially performed by the 2-lobe technique with initial 5 and 7 o’clock incisions, including the median in one of the incisions to be enucleated with the corresponding lateral lobe. The other lobe was then enucleated separately. Gradually, a switch over to the newer en bloc enucleation of the prostate was done, in which the prostate was enucleated as a single lobe to be dropped in the bladder, followed by morcellation. Limited studies have compared the standard 2-lobe technique with the en bloc technique. In light of this context, the current study aimed to compare the safety and efficacy of en bloc and two-lobe techniques of HoLEP, focusing mainly on the incidence of postoperative urinary incontinence.

## Material and Methods

### Study Design

This was a prospective, randomized study conducted on patients with benign prostatic obstruction who underwent HoLEP between September 2020 and March 2021. The study was approved by the MGM Medical college Institutional Ethics Committee (Approval No: MGM-ECRHS/2020/37) and the study procedure was in accordance with the principles of the Declaration of Helsinki. Written informed consent was obtained from all study patients before enrollment.

### Eligibility Criteria

Patients aged 50 and above, those having refractory lower urinary tract symptoms secondary to BPH, size of the gland being 50-150 g, an international prostate symptom score (IPSS) > 15, and those willing to give informed consent were included in the study. Patients with prostate cancer, urethral strictures, previous prostate surgery, bladder stones, and neurogenic bladders were excluded from the study. All patients underwent preoperative evaluation with abdominal sonography for prostate evaluation, including weight, prostate-specific antigen, and IPSS.

### Study Procedure

All patients were randomly assigned in a 1:1 ratio to receive either en bloc or 2-lobe technique. Randomization was done using an online randomizer tool (https://www.randomizer.org/).

For HoLEP, a VersaPulse Power Suite laser (Lumenis, Yokneam, Israel) with a wavelength of 2140 nm and a 550 µm end firing laser fiber was used, with the power being set at 100 W (2 J × 50 Hz) during enucleation and 25 W (1 J × 25 Hz) during hemostasis. The procedure used a 26 Ch resectoscope (Karl Storz, Tutlingen, Germany) with continuous saline irrigation. All procedures were performed by 1 surgeon (ADM) experienced in EEP.

The 2-lobe technique began with a floor incision at 6 o’clock on the bladder neck toward the verumontanum until the circular capsular fibers were visualized. At the verumontanum, the incision was continued counterclockwise, laterally from 5 o’clock to 3 o’clock. Then, at 12 o’clock, an incision was made from the neck of the bladder toward the verumontanum. The 12 o’clock incision was then brought down until it connected with the 3 o’clock incision. The left lobe was then completely enucleated until the bladder neck fibers were reached. The enucleated left lobe was displaced in the bladder. The same technique was used for right-lobe enucleation.

During the en bloc technique, an incision was made at the left lateral side of the verumontanum until the capsule was reached. A similar incision was then taken on the right side of the verumontanum to reach the capsule. Then a circumferential mucosal incision was marked on the prostatic mucosa just in front of the verumontanum. This was the early apical release incision. Both the apical lobe incisions were then connected with the floor incision just in front of the verumontanum. The left lateral lobe was then enucleated laterally until the 3 o’clock position from the 6 o’clock one. Similar enucleation was done from the 6 o’clock position till the 9 o’clock position. The roof incision at 12 o’clock was deepened till the capsule and enucleation were continued on either side till 10 o’clock on the right side and 2 o’clock on the left lobe of the prostate. The enucleation was then continued in a circular fashion until most of the gland was enucleated. The bladder neck was then opened, and the 12 o’clock position and the incision were brought down to the base of the prostate so that the gland was free from all sides. The floor tissue was enucleated at the end to drop the single lobe of the prostate in the bladder (Figure 2). Orifices were visualized and protected at all times.

Morcellation was done using the Versacut morcellator (Lumenis, Yokneam, Israel). The enucleation time and the morcellation time were noted. Postoperatively, a 22F 3-lumen Foley’s catheter was placed, and irrigation started. The catheter was removed on the second postoperative day. Patients were followed up for 3 months, and postoperative urinary incontinence (stress and urge), if any, were evaluated. IPSS and postoperative maximum flow rate (Q_max_) were assessed.

### Endpoints

The primary endpoint was efficacy in terms of enucleation and surgery time for both enucleation techniques. The secondary endpoint was to evaluate the complications, especially with regard to urinary incontinence.

### Statistical Analysis

All statistical analyses were performed using Statistical Package for Social Science Statistics software, version 23.0 (IBM SPSS Corp.; Armonk, NY, USA). The qualitative data were expressed as numbers and proportions, while the quantitative data were expressed as means. Categorical and continuous variables were compared with the chi-square test and Mann–Whitney *U*-test, respectively. Statistical significance was defined as *P* < .05.

## Results

**Figure 1.**
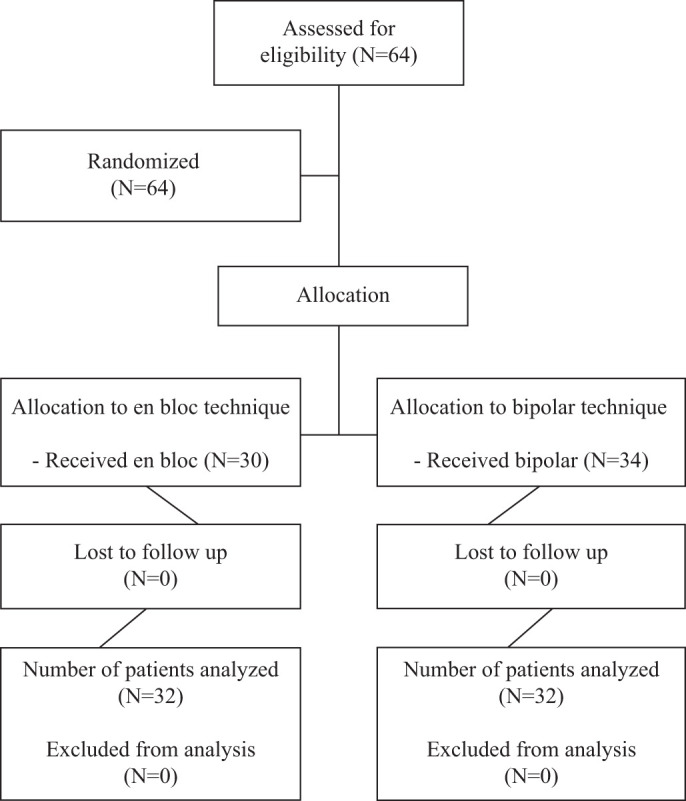
Consort flow diagram.

**Figure 2.**
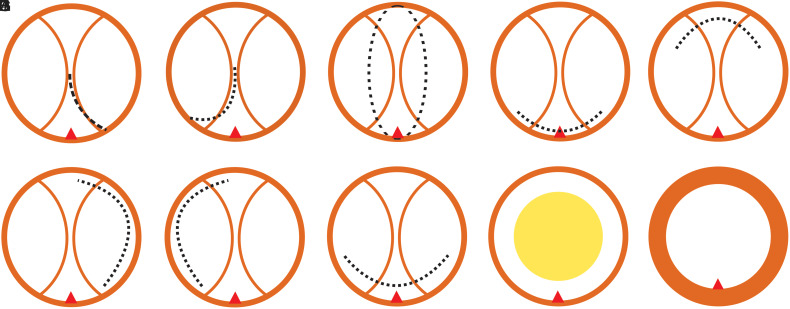
Surgical steps of en bloc enucleation of prostate. A: Left apical incision. B: Right apical incision. C: Circumferential apical mucosal release incision. D: Floor incision deepened from 5 O’clock to 7 O’clock. E: Roof incision deepened from 10 O’clock to 2 O’clock and bladder neck opened. F: Left lateral lobe dissected. G: Right lateral lobe dissected. H: Floor incision deepened till the bladder neck is opened. I: En bloc enucleation done. J: Intact sphincter.

In this study, 64 patients who underwent HoLEP either by en bloc (n = 30) or 2-lobe (n = 34) were included. All patients were eligible for data analysis (Figure 1). The mean age was comparable in both groups (68.53 vs. 67.55 years; *P* = .62). Preoperative serum creatinine (1.08 mg/dL vs. 1.20 mg/dL, *P* = .35), serum PSA (3.78 vs. 4.63 ng/mL; *P* = .29), and mean prostate volume (79.43 g vs. 79.88 g; *P* = .92) were comparable in both groups. Hypertension was the most common comorbidity observed in both groups (Table 1).

The enucleation time was significantly shorter in the en bloc group compared to the 2-lobe group (54.20 vs. 61.67 minutes; *P* = .03). The morcellation time was comparable between the groups (*P* = .87). The mean operative time was found to be shorter in the en bloc group compared to patients undergoing the 2-lobe technique (72.36 vs. 80.50 minutes; *P* = .03) (Table 2). However, this difference did not reach statistical significance.

Intraoperatively, there was 1 patient with extravasation due to a small bladder perforation during morcellation in the en bloc group. Tapping was done intraoperatively and then managed conservatively, leading to complete absorption. Hematuria and clot retention were observed in 1 patient in each group. Clot retention was managed by bladder wash in both groups. Three patients had Calvien–Dindo grade I complications in each group (Table2).

In both groups, there was no significant difference in preoperative and postoperative parameters like a drop in hemoglobin, IPSS score, and Q_max_ (Table 3). There was a significant difference in the improvement in the QoL score in the en bloc group as compared to the 2-lobe group (3.80 vs. 2.11; *P* < .01).

Postoperatively, the occurrence of stress urinary incontinence was considerably higher in the 2-lobe group compared to the en bloc group on day 1 (*P* < 0001), day 7 (*P* = .02), and day 30 (*P* = .003). However, the rates of total incontinence and urge incontinence in both groups were comparable (Table 4).

## Discussion

In the present study, the clinical outcomes of 2 different surgical approaches for HoLEP, including the conventional 2-lobe technique and the en bloc technique, were compared. It was observed that the en bloc technique shortened the enucleation time more than the conventional 2-lobe technique. The en bloc technique witnessed a significantly lower incidence of stress urinary incontinence than the 2-lobe technique.

The basic principle of endoscopic enucleation was to resemble or mimic adenoma enucleation along the anatomically correct surgical capsule as done in open prostatectomy.^15^ In 1986, Hiraoka et al^16^ conducted the first endoscopic enucleation with monopolar cautery, but due to technical imperfections it failed to propagate the technique, mainly due to the use of monopolar loops. In 1998, enucleation of the prostate became prominent when Gilling et al^6^ introduced the first HoLEP procedure with a morcellator to cut and evacuate the lobes. The Gilling (or 3-lobe) technique is the most used technique today. Baazeem et al^17^ described a 2-lobe technique with blunt dissection with the resectoscope beak to simplify EEP. They estimated that around 20 cases are required to gain experience with the HoLEP procedure. Later, multiple papers were published with different alterations to the en bloc technique of EEP and its advantages. New EEP techniques were formulated mainly to facilitate learning; this principle was consistent with Scoffone et al’s^18^ study, which proposed the en bloc no-touch HoLEP technique to enhance the learning process. The primary distinction was the initial recognition of the enucleation plane at the verumontanum and the continuous horse-shoe enucleation of all lobes simultaneously. They claimed that since one of the challenging aspects of surgical plane identification only needed to be done once, it might reduce operative time and promote learning. Castellani et al^19^ highlighted that en bloc EEP, as compared to the standard 3-lobe technique, can be done with ease by a novice. This present study made it quite evident that the careful understanding of prostate anatomy during the en bloc technique makes it challenging at the initial steps of learning.

A study by Saredi et al^4^ concluded that the en bloc lowers the operative time, blood loss, and energy when compared to the standard 3-lobe procedure. These results were similar to the observations in Ito et al, who observed shorter operative time and enhanced superior enucleation efficiency in HoLEP by a complete en bloc procedure when compared to the 3-lobe technique.^12^ This study also showed that the mean operative time (80.5 vs. 72.36 minutes; *P* = .03) was comparatively higher in the 2-lobe group than in the en bloc group. However, the enucleation time (61.67 vs. 54.2 minutes; *P* = .03) was significantly higher in the 2-lobe group as compared to the en bloc group. The morcellation time in both groups was comparable. In contrast to this, Enikeev et al^20^ reported that there was no difference between the 2-lobe and HoLEP techniques in terms of enucleation duration (68.81 vs. 67.41 minutes; *P* = .604), and that in major glands, the morcellation time was shorter in the en bloc group compared to the 2-lobe group. In this study, we attribute the shortened operative and enucleation times to a single circumferential incision at the apex, which results in obtaining the surgical plane only once, as compared to the 2-lobe technique, wherein the surgical planes must be reached separately for both lobes.

As reported by multiple studies, the incidence rate of transient incontinence was 1.4% to 44% in patients who underwent this procedure.^21-24^ In the present study, the incidence of stress urinary incontinence was considerably higher in the 2-lobe group as compared to the en bloc group, indicating that the en bloc technique is efficient in preserving the external sphincter. In a prospective analysis of 168 patients by Tuccio et al^25^, it was concluded that post-HoLEP, urinary stress was significantly reduced in the en bloc group compared with the traditional 3-lobe technique. Another study by Gong et al^26^ observed that post-modified HoLEP in patients with BPH resulted in less postoperative transient stress incontinence in 3 out of 189 patients, with spontaneous recovery within 3 months. A retrospective study used a novel en bloc HoLEP technique with anteroposterior dissection, which resulted in a low rate of stress urinary incontinence (2.7%) at 2 weeks postoperatively.^27^ Chun Li et al^28^ proposed a novel technique of 3 horseshoe-shaped incisions for EEP, which resulted in less transient urinary incontinence. Saitta et al^13^ thoroughly described the stepwise en bloc enucleation procedure with an early apical mucosal release technique, which resulted in less stress urinary incontinence.

This study compared all the types of incontinence expected after EEP—urge, stress, and total incontinence. No patients had urge or total incontinence after 30 days in both groups. However, the incidence of stress urinary incontinence was significantly less with the en bloc technique than with the 2-lobe technique; this result was consistent with multiple studies as mentioned above. This can be attributed mainly to 2factors: firstly, the circumferential early apical mucosal release incision during the en bloc technique, which disconnects the sphincter from the adenoma in the early intervention stage. And secondly, we avoided the enucleation of the adenoma by mechanical dissection of the resectoscope beak, which can lead to additional traction on the sphincter fibers. The adenoma was lifted by the resectoscope beak to see the junction between the adenoma and the prostatic capsule.

This study’s primary limitations were the small sample size and the short duration of both groups’ evaluations of patients. To demonstrate that the en bloc procedure is preferable over the traditional EEP techniques, further data with long-term follow-up is required. Another limitation was that these were the early surgeries performed by the en bloc enucleation technique, after excluding a few initial operations performed by both methods.

En bloc and 2-lobe techniques of HoLEP showed similar postoperative outcomes in terms of improvement in Qmax and drop in hemoglobin. However, the enucleation time, operative time, and postoperative stress incontinence after en bloc enucleation of the prostate were significantly less as compared to the 2-lobe technique. Also, the QoL score was significantly better with the en bloc technique. Therefore, the study concludes that the en bloc technique could be a better choice for all patients undergoing HoLEP for EEP. However, more extensive research is required to confirm these findings before making definitive recommendations.

## Figures and Tables

**Table 1.** Preoperative Patient Data

	En bloc (n = 30)	2 lobe (n = 34)	*P*
Age (years) mean (SD)	68.53 ± 7.21	67.55 ± 8.43	.62
Comorbidities n (%)	3	3	3
Cardiovascular disease	4 (13.33)	2 (5.88)	.31
Hypertension	12 (40)	10 (29.4)	.35
Type 2 diabetes mellitus	2 (6.67)	8 (23.5)	.059
COPD	-	3 (8.82)	-
Hypothyroidism	-	2 (5.88)	-
Serum Creatinine (mg/dL)	1.08 ± 0.30	1.20 ± 0.67	.35
Serum PSA (ng/mL)	3.78 ± 3.15	4.63 ± 3.19	.29
Prostate weight (g)	79.43 ± 21.79	79.88 ± 18.38	.92

**Table 2.** Comparison of Intraoperative Parameters and Complications

	En bloc (n = 30)	2 lobe (n = 34)	*P*
Enucleation time (minutes)	54.20 ± 14.24	61.67 ± 12.82	.03
Morcellation time (minutes)	19 ± 3.63	18.85 ± 3.60	.87
Total operative time (minutes)	72.36 ± 15.30	80.5 ± 15.35	.03
Extravasation	1 (3.33)	0	3
Transurethral resection syndrome/fluid absorption	0	0	3
Hematuria	1 (3.33)	1 (2.94)	.92
Hematuria requiring transfusion	0	0	3
Clot retention	1 (3.33)	1 (2.94)	.92
Fever	2 (6.67)	3 (8.82)	.75
Clavien–Dindo classification (grade I)	3 (10)	3 (8.82)	.87

Data shown as mean (SD).

**Table 3.** Comparison of Preoperative and Postoperative Parameters

	En Bloc (n = 30)	2 Lobe (n = 34)	*P* En Bloc vs. 2 Lobe
Pre-op Q_max_ (mL/s)	7.14 ± 2.40	6.82 ± 2.96	.64
Post-op Q_max_ (mL/s)	17.84 ± 2.67	17.87 ± 2.91	.96
Difference in pre- and post-op Q_max_3	−10.70 ± 1.48	−11.04 ± 1.64	.38
Pre-op Hb (g/dL)	12.51 ± 1.79	12.79 ± 1.67	.52
Post-op Hb (g/dL)	11.61 ± 1.43	11.89 ± 1.66	.47
Difference in pre- and post-op Hb (g/dL)	0.9 ± 0.25^*^3	0.89 ± 0.24^*^3	.87
Pre-op IPSS	24.73 ± 4.69	25.32 ± 4.45	.60
Post-op IPSS	8.63 ± 4.17	8.38 ± 3.97	.80
Difference in pre and post-op IPSS	16.1 ± 2^*^3	16.94 ± 2.1^*^3	.10
Pre-op QOL	6.03 ± 9.29	4.61 ± 0.98	.38
Post-op QOL	2.23 ± 1.96	2.50 ± 3.35	.70
Difference in pre- and post-op QOL	3.80 ± 3.63^#^3	2.11 ± 1.19^**^3	.01

Data shown as mean (SD). Pre and post- operative differences.

Hb, hemoglobin; IPSS, international prostate symptom score; QOL, quality of life.

*P < .0001. **P < .007. #*P* < .04.

**Table 4.** Comparison of Postoperative Incontinence

	En bloc (n = 30)	Two-lobe (n = 34)	*P*
**Total Incontinence**3	3		
Day 1	0	0	-
Day 7	0	0	-
Day 30	0	0	-
**U** **rge Incontinence**3	3		
Day 1	30 (100)	34 (100)	3
Day 7	6 (20)	13 (38.2)	.11
Day 30	0	0	-
Stress Incontinence	3		
Day 1	3 (10)	22 (64.7)	<.0001
Day 7	1 (3.33)	8 (23.5)	.02
Day 30	1 (3.33)	11 (32.4)	.003
